# BAT: Bisulfite Analysis Toolkit: BAT is a toolkit to analyze DNA methylation sequencing data accurately and reproducibly. It covers standard processing and analysis steps from raw read mapping up to annotation data integration and calculation of correlating DMRs.

**DOI:** 10.12688/f1000research.12302.1

**Published:** 2017-08-16

**Authors:** Helene Kretzmer, Christian Otto, Steve Hoffmann

**Affiliations:** 1 Bioinformatics Group, Department of Computer Science, and Interdisciplinary Center for Bioinformatics, University of Leipzig, Leipzig, 04109, Germany; 2 Transcriptome Bioinformatics, Research Center for Civilization Diseases (LIFE), University of Leipzig, Leipzig, 04109, Germany; 3 ecSeq GmbH, Leipzig, 04275, Germany

**Keywords:** DNA methylation, epigenetics, bisulfite sequencing, WGBS, RRBS, software, DMRs, integrative analysis

## Abstract

Here, we present **BAT**, a modular bisulfite analysis toolkit, that facilitates the analysis of bisulfite sequencing data. It covers the essential analysis steps of read alignment, quality control, extraction of methylation information, and calling of differentially methylated regions, as well as biologically relevant downstream analyses, such as data integration with gene expression, histone modification data, or transcription factor binding site annotation.

## Introduction

High-throughput DNA methylation sequencing protocols, such as whole-genome bisulfite sequencing (WGBS) and targeted bisulfite sequencing (e. g., RRBS), have made it possible to precisely and accurately measure this major epigenetic modification on a genome wide scale. The impact of DNA methylation on processes, such as cell differentiation, gene expression, chromatin structure, and cancerogenesis, has raised substantial interest in analyzing DNA methylation in many sectors of life sciences. For example, methylomes of a large number of samples have been sequenced in the context of cancer projects and developmental studies ^1– 5^. Also researchers investigating obesity, neurodegenerative diseases, Alzheimer’s, or Parkinson’s disease, have begun to focus on DNA methylation ^6– 9^.

A number of time consuming data analysis steps are required in virtually all these projects, i. e., quality control, read alignment, and methylation rate calculation. However, performing each step by hand is highly error prone, takes time, and impacts reproducibility. To ensure a consistent and reproducible processing, we have developed the Bisulfite Analysis Tooklit BAT. The workflow enables a fast and easy analysis of bisulfite converted high-throughput sequencing reads. It is specifically designed to facilitate the analysis for biologists and physicians with little bioinformatic knowledge, as well as for bioinformaticians that already work on sequencing data, but are not familiar with the characteristics of bisulfite sequencing data.

## Methods

**Figure 1.**
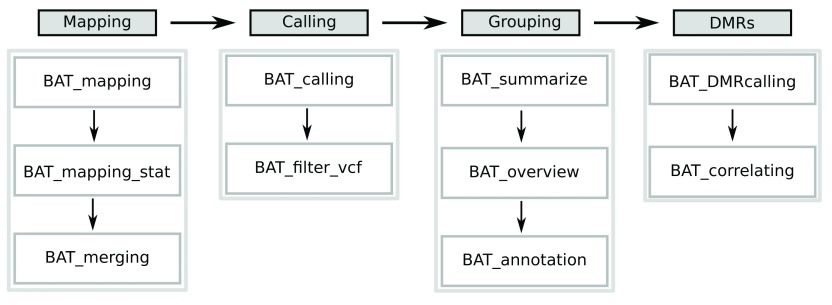
BAT workflow. It comprises four modules covering (left to right) read alignment, methylation rate calling, basic group analysis, and DMR calling. The modules consist of a collection of scripts that build up on one another, but easily single steps can be covered by alternative tools.

BAT is a modular toolkit allowing to easily generate workflows to analyze bisulfite sequencing data. The toolkit includes modules for read alignment (mapping module), methylation level estimation (calling module), grouping of samples (grouping module) and identification of differentially methylated regions (DMR module) ( Figure 1). Further modules allow the integration of gene expression, histone modification data, or transcription factor binding site annotation. These modules facilitate the functional analysis of the effects of differential methylation.

Each of the modules can be run on its own, and the minimal system requirements depend on the respective module. The computational most expensive module is the mapping module. Here, the aligner segemehl ^10^ in its bisulfite mode is used, which requires about 55 GB physical RAM for the alignment of reads to the human genome hg19.

The toolkit itself is written in Perl and calls software components mainly written in C and R to ensure swift calculations. All software requirements are listed on our website ( www.bioinf.uni-leipzig.de/Software/BAT/install/#requirements). The default parameters for the tools included in the BAT pipeline are optimized to process bisulfite sequencing data for most applications. In order to enhance reproducibility and reduce potential errors, the number of parameters that need to be set by the user has been carefully reduced to a minimum. Due to its modularity, however, the toolkit is flexible and can easily be extended or customized to specific needs. To allow for workflow modifications and extensions, standardized formats are used and interfaces to several other tools are provided. Basic steps, e.g., processing from raw reads to a single alignment file from multiple sequencing runs, is split into its pre-, post-, and main processing steps, to allow for the customized extension of the workflow. Error handling is eased by parameter and file checks prior to the analysis, and meaningful error messages allow a quick trouble shooting.

A detailed documentation of all modules, including parameter description, recommended additional tools, analysis reports, and data visualizations produced by the BAT workflow are summarized on www.bioinf.uni-leipzig.de/Software/BAT. Moreover, all automatically created visualizations are shown on the webpage. Data and figures displayed there are derived from a small example data set of two groups with four samples each, adopted from Kretzmer *et al* ^11^. Our webpage provides raw FASTQ files of one sample as well as the methylation rate files of all eight samples along with expression and annotation data. This example data set and shell scripts covering all modules of BAT can be downloaded and adapted together with the toolkit.

Furthermore, BAT is provided as Docker ^12^ image and can be obtained from https://hub.docker.com/r/christianbioinf/bat/. The Docker images ensure platform independent usage of our toolkit. All programs that are used by BAT are already installed in the Docker image and dependencies are resolved. Existing hard drives are mounted to avoid time consuming translocation or upload of the frequently huge data.

## Use cases

Resembling a common study design, we assembled a small case-control example dataset, adopted from recently published data ^11^. It is a subset of a paired-end human WGBS dataset, comprising 8 samples (control: S1–S4, case: S5–S8). It comprises the raw reads in FASTQ format of one sample and the already called methylation rates of all 8 samples in VCF format. The following modules can now be used to process and analyze bisulfite sequencing data including detection of methylation differences between case and control samples. The use case starts with the alignment of the raw sequencing data using the mapping module. The single components of BAT and their functionality are described in the following:

### Mapping

The read alignment step is taken care of by the module BAT_mapping. It includes a bisulfite-sensitive read alignment using segemehl ^10^, a quality filtering step, and the conversion of the alignments to an indexed and compressed BAM file by samtools ^13^. Using BAT_mapping_stat, the quality of the mapping can be assessed by the number and fraction of mapped pairs or reads, the multiplicity of read alignments, and the alignments’ error rates. In case of large experiments where a sample is sequenced multiplexed on multiple lanes or flow cells, the read alignments of each sample can easily be merged using BAT_merging, including the addition of read group information to allow for tracebacks of lane effects if necessary.

### Calling

**Figure 2.**
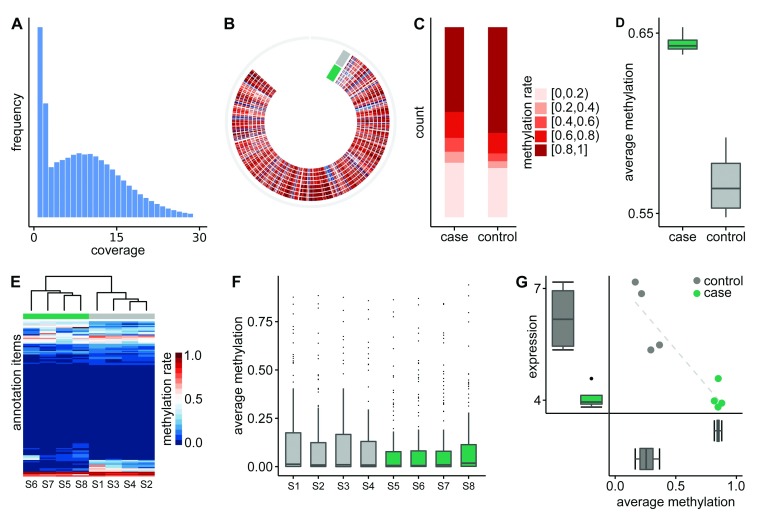
Figure 2. Selection of figures generated on-the-fly by BAT during the analysis of the example dataset. Annotation items are ENCODE transcription factor binding sites for GM12878 cell line. **A**) Distribution of coverage. **B**) Circos plot showing the genome-wide methylation level of eight samples as heatmap. **C**) Binned distribution of average methylation rate per CpG for each group. **D**) Boxplots of genome-wide mean methylation rate per group. **E**) Hierarchical clustered heatmap of the methylation rates of all samples over all annotation items. **F**) Boxplots of average methylation rate per annotation item. **G**) Correlating DMR plot shows methylation and expression of a DMR - gene pair. Note that all figures were produced by BAT itself, but were minorly post-edited to fit the limited space.

Following mapping, the methylation information needs to be extracted from the read alignments. Prior to this methylation calling it is, however, recommended to exclude potential biases by clipping alignment overlaps of paired-end reads (e.g. using bamutil’s clipoverlap ^14^) or by excluding incompletely converted or artificially introduced cytosines with the M-bias detection method (e.g. using BSeQC ^15^). Subsequently, the methylation information can be extracted using the module BAT_calling, which returns a VCF-style file that includes detailed information for each cytosine. This initial set of positions can be filtered by coverage using BAT_filtering to exclude unreliable methylation information from either lowly covered or very highly covered positions (e.g. in repetitive regions). Moreover, it is also possible to filter by genomic context (e.g., to restrict to CG context only). Apart from a VCF file, BAT_filtering reports the methylation level at positions passing the filter in bedGraph format for easy inspection in IGV ^16^ or upload to the UCSC genome browser ^17^. Additionally, the module automatically produces plots showing the distribution of coverages and methylation rates for the complete and the filtered set of positions ( Figure 2A), giving the user the opportunity to check and possibly fine tune the filtering parameters.

### Groups

The third module now facilitates the transition from single sample analysis to groups of multiple samples. First, methylation information from individual samples is combined to groups and summarized with BAT_summarize. It reports the mean methylation rate per group and position as well as difference of the group’s mean methylation rate per position. The summary module can be parameterized to only report positions where each group has a minimum number of samples with sufficient coverage. For convenience, all files are exported in both bedGraph and bigWig format for inspection in UCSC genome browser or IGV. Moreover, a circos plot containing a genome-wide methylation rate heatmap for each sample is automatically produced ( Figure 2B). Based on the summary files, a number of overview statistics and plots can be generated using BAT_overview. This includes a hierarchical clustering of the samples based on their methylation profile, a plot of binned mean methylation rates per group ( Figure 2C), boxplots of group-wise mean methylation rates ( Figure 2D), a smoothed scatterplot showing the correlation between the groups’ mean methylation rate per position, and a barplot of the distribution of group methylation differences. Subsequently, BAT_annotation can be used to inspect the methylation of the samples in regions of interest or annotations such as transcription factor binding sites (TFBS), CpG islands, shores, or promoter regions. Therefore, a hierarchically clustered heatmap of all samples ( Figure 2E), is produced and the per-group and per-sample mean methylation rate is calculated ( Figure 2F).

### Differential methylated regions

Finally, the fourth module features the identification and analysis of differentially methylated regions (DMRs) between groups ( BAT_DMRcalling). It employs the DMR calling tool metilene ^18^ which is based on circular binary segmentation of the group methylation difference signal in conjunction with a two-dimensional non-parametric statistical test. Afterwards, the DMRs reported by metilene can be filtered by several criteria, e.g., length (in nt or number of Cs), significance (i.e., q-value), and minimum mean methylation difference, and then converted to BED/bedGraph format. The BED file contains unique identifiers per DMR and reports regions of hyper/hypo methylation. Additionally, the bedGraph file can be used to display the mean group methylation difference of the DMRs. Moreover, BAT_DMRcalling produces overview statistics of the set of filtered DMRs including a histogram of the length and methylation difference of the filtered DMRs, a correlation plot of the mean methylation rate of DMRs in both groups and a plot of the methylation difference vs. the q-value for each DMR. Last but not least, BAT_correlating allows for integration of the DMRs with expression data. Given the methylation information, an expression value of genes, and an association between DMRs and genes, the correlation between both types of data can be examined in order to find correlating DMRs (cDMRs). For each DMR-gene pair, a linear and non-linear correlation coefficient is calculated and a correlation plot ( Figure 2G), showing methylation and expression of each sample, is generated.

## Summary

BAT has already successfully been applied in the framework of a large cancer genome study, the ICGC MMML-Seq ^11^. The streamlined processing and analysis modules improve and accelerate the analysis by reducing hands on time and user errors. The modularity of BAT, as well as its input and output formats, allow to easily extend or customize the default workflows. For instance, it is possible to easily integrate tools such as BisSNP ^19^ or BS-Snper ^20^ or DMR calling tools.

The custom visualizations of the methylation data facilitate data mining and allow to inspect the data quality at each step of the analysis. This is necessary to increase the chance of an early detection of errors, e.g., in library preparation and data handling. Therefore, quality control statistics and graphics are produced continually throughout the entire pipeline.

Taken together, BAT is a collection of modular steps for analyzing bisulfite sequencing data that (i) can easily be run on various platforms due to the virtualization via Docker, (ii) can be combined with or extended by other tools, (iii) automatically generates publication-ready graphics, and (iv) supports data integration, e.g., annotation or gene expression data.

## Software and data availability

Software available from: www.bioinf.uni-leipzig.de/Software/BAT/download

Source code available from: https://github.com/helenebioinf/BAT

Archived source code as at time of publication: http://doi.org/10.5281/zenodo.838200 ^21^.

License: MIT

Example data available from: www.bioinf.uni-leipzig.de/Software/BAT/download/#example_data
