# Supplementary material for: BAT: Bisulfite Analysis Toolkit: BAT is a toolkit to analyze DNA methylation sequencing data accurately and reproducibly. It covers standard processing and analysis steps from raw read mapping up to annotation data integration and calculation of correlating DMRs
Source: F1000Res. 2017 Aug 16;6:1490. [Version 1] doi: 10.12688/f1000research.12302.1 (PMC5590080; doi:10.12688/f1000research.12302.1)
Supplement: Peer review history

# Peer review: Referee response for version 1

**DOI:** 10.5256/f1000research.13317.r25075

**Published:** 2017-09-12

**Authors:** Lars Feuerbach

**Affiliations:** 1 Comparative Cancer Genomics, Division of Applied Bioinformatics, DKFZ (German Cancer Research Center), Heidelberg, Germany

The manuscript “BAT: Bisulfite Analysis Toolkit” presents a software pipeline for the analysis of sequencing-based analysis of bisulfite treated DNA. It introduces the major modules of this pipeline and familiarize the reader with  their basic function, compatibilities and output, but is obviously not intended to provide sufficient detail to allow reimplementation of the described modules. Instead, it refers to external resources such as research articles and documentary webpages, which provide most of this information.

 The article excels in providing a researcher who has to choose among several software pipelines for his next methylation project with the necessary information on BAT, without attempting to benchmark it against other approaches.

 Especially, the offer of a dockerized pipeline version and a real example datasets ensures the applicability of the software, while simultaneously proving the claim of improved reproducibility.

 Another prominent claim, namely the compatibility with other modules for instance alternatives to segemehl, is less well documented. Here the article would profit from an extended example in which some of the modules are exchanged by third party alternatives, e.g. in the alignment step or during the grouping.

 Finally, the authors describe the utility of their diagnostic diagrams depicted in figure 2 for the detection of quality problems. To this end a supplementary figure/resource in which a number of examples of how several quality problems manifest in these diagrams is required, not only to proof this statement, but also to educate less experienced users.

 Minor comment:

 The sentences “However, performing each step by hand is highly error prone, takes time, and impacts reproducibility” in the introduction is formulated unfavorably, as it can be misread as a suggestion that someone would attempt to analyze a WGBS dataset by hand.

I have read this submission. I believe that I have an appropriate level of expertise to confirm that it is of an acceptable scientific standard.

# Peer review: Referee response for version 1

**DOI:** 10.5256/f1000research.13317.r25078

**Published:** 2017-09-06

**Authors:** Ishaan Gupta

**Affiliations:** 1 Brain and Mind Research Institute, Weill Cornell Medicine, New York City, NY, USA

BAT: Bisulphite analysis toolkit is a timely software which provides an end-to-end solution for performing DNA methylation analysis. The toolkit follows "good software practises" and has a clearly laid out work flows, efficient code , extensive documentation and has limited dependencies. Further, ability to perform the complete analysis from sequencing data to actual interpretation and integration of data as shown in the example data in the toolkit from the manuscript by the same authors "DNA methylome analysis in Burkitt  and follicular lymphomas identifies differentially methylated regions linked to somatic mutation and transcriptional control" ^1^  suggests that the method implemented in the toolkit for calling differentially methylated  regions (DMRs) is not only much faster than existing solutions but also extracts biologically relevant information about methylation.

 I outline my reasons below :

- Is the rationale for developing the new software tool clearly explained?

The rationale for developing the software well explained as sequencing data especially bisulphite sequencing data are prone to human errors and increasing number of samples being processed for cohorts tackling complex disease phenotypes warrant for a streamlined reproducible workflows. Also the ease of use is a term often loosely used for many bioinformatics tools are under-appreciated by the community but the authors have done well here by providing a docker image that obviates any platform dependencies to provide an out of the box solution. 

 Suggestion:

 As a rationale it would would great if the authors could add a few lines on their method of calculating DMRs in the introduction to contrast with existing tools, I believe this would enhance the manuscript and further convince the readers to use this tool-kit.

  

-  Is the description of the software tool technically sound?

Software documentation is thorough and technically sound. 

 Moreover, Dr. Hoffmann's lab has been quite consistent in releasing regular updates for their previous tools and is responsive to bug-reports.

 Suggestion:

 It is accurate that segemehl requires 55GB to align the entire human genome but it would be important to also point out that alignment could be run on individual chromosomes separately and then combined later which significantly reduces this memory intensive step.

-  Are sufficient details of the code, methods and analysis (if applicable) provided to allow replication of the software development and its use by others?

The workflows are well laid out and broken down into the individual modules establishing a replicable software design. Each module can be run individually or together through the perl wrapper and come with appropriate description of flags used in the command line help allowing a look under the hood of the code. Further, each tool is well documented and the code is commented making the tool reproducible.

-  Is sufficient information provided to allow interpretation of the expected output datasets and any results generated using the tool?

The example provided herewith runs well and one can quickly reproduce the plots from Kretzmer *et al* 2015 ^1^.

I have read this submission. I believe that I have an appropriate level of expertise to confirm that it is of an acceptable scientific standard.

## References

[1] KretzmerHBernhartSHWangWHaakeAWenigerMABergmannAKBettsMJCarrillo-de-Santa-PauEDooseGGutweinJRichterJHovestadtVHuangBRicoDJühlingFKolarovaJLuQOttoCWagenerRArnoldsJBurkhardtBClaviezADrexlerHGEberthSEilsRFlicekPHaasSHummeMKarschDKerstensHHDKlapperWKreuzMLawerenzCLenzekDLoefflerMLópezCMacLeodRAFMartensJHAKulisMMartín-SuberoJIMöllerPNageIPicelliSVaterIRohdeMRosenstielPRosolowskiMRussellRBSchilhabelMSchlesnerMStadlerPFSzczepanowskiMTrümperLStunnenbergHGKüppersRAmmerpohlOLichterPSiebertRHoffmannSRadlwimmerB: DNA methylome analysis in Burkitt and follicular lymphomas identifies differentially methylated regions linked to somatic mutation and transcriptional control. *Nat Genet*.2015;47(11) : 10.1038/ng.3413 1316-1325 10.1038/ng.3413 26437030PMC5444523

# Peer review: Referee response for version 1

**DOI:** 10.5256/f1000research.13317.r25076

**Published:** 2017-08-29

**Authors:** Bob Zimmermann

**Affiliations:** 1 Department of Molecular Evolution and Development, University of Vienna, Vienna, Austria

This article presents a tool aggregate which can be a useful one-stop-shop and/or starting off point for analyzing bisulfite data. The authors detail the package and demonstrate its usefulness in an example analysis. Most of the information needed to decide whether to use this package is contained in the article.

 A notable exception are the "further modules" mentioned in the first paragraph of the Methods section. While it is clearly useful to integrate gene expression, histone modification data and transcription factor binding site information to your analysis, the reader cannot get an impression of whether the package does this effectively. It would be useful to include either an expansion on this topic or better yet to add example analysis with these modules as well, space permitting. If the authors are space confined, it would be useful to point to where an example of this can be found on the web, as I was unable to locate it on the project page.

 A critical omission from the introduction is a short technical background on bisulfite sequencing and its analysis. The reader has no basis to understand why a "VCF-style file that includes detailed information for each cytosine" (in the Calling subsection) would be useful.

 Some minor issues were:

- the phrase "grouping of samples" in the second sentence of the Methods section does not really clarify anything about the function of the grouping module. I would suggest to use "sample group analysis"
- "Due to its modularity, however" is awkwardly worded and could be better expressed as "The toolkit's modularity makes it flexible, extensible and customizable for users with specific needs".
- the sentence "Basic steps, e.g. ..." should say "are" instead of "is".
- "Resembling a common study design," in the Use cases section does not express what I believe is the authors' intended meaning, and could be better worded as "In order to illustrate the results of using our toolkit on a common study design,"

I have read this submission. I believe that I have an appropriate level of expertise to confirm that it is of an acceptable scientific standard.
